# Proposal of a bioinformatics approach to predict molecular mechanisms involved in inflammatory response: case of ATRA and 1,25(OH)_2_D in adipocytes

**DOI:** 10.1080/15592294.2023.2201516

**Published:** 2023-04-18

**Authors:** Esma Karkeni, Thomas Payet, Julien Astier, Flavie Sicard, Lourdes Mounien, Jean-François Landrier

**Affiliations:** a Aix-Marseille Université, C2VN, INRAE, INSERM, Marseille, France; b PhenoMars, C2VN, INRAE, INSERM, CriBiom, Marseille, France

**Keywords:** NF-κB, bioinformatics, adipocytes, adipose tissue, vitamins, vitamin a, vitamin D

## Abstract

Several inflammatory markers such as cytokines, chemokines, and microRNAs (miRNAs) are well known to be induced during obesity and are strongly linked to their comorbidities. Among many others factors, the micronutrient status is suspected to reduce obesity-associated inflammation via blunting inflammatory signalling pathways. This is notably the case for active forms of vitamin A (all-trans retinoic acid ATRA) and vitamin D (1,25(OH)_2_D) as previously shown. In the present study, we aimed to implement a new bioinformatics approach to unveil commonly regulated signalling pathways through a combination of gene and miRNA expression sets impacted by ATRA and 1,25(OH)_2_D in adipocytes. In a first set of experiments, we focused only our attention on ATRA and demonstrated that it reduced LPS-mediated miRNA expression (miR-146a, miR-150, and miR-155) in mouse adipose tissue, in adipocyte cultures, and in adipocyte-derived vesicles. This result was confirmed in TNFα-induced miRNA in human adipocytes. Then, bioinformatic analysis highlighted that both ATRA and 1,25(OH)_2_D-regulated genes and miRNA converge to the canonical ‘nuclear factor Kappa B (NF-κB) signalling pathway.’ Altogether, these results showed that ATRA has anti-inflammatory effects on miRNA expression. In addition, the proposed bioinformatic model converges to NF-κB signalling pathway that has been previously demonstrated to be regulated by ATRA and 1,25(OH)_2_D, thus confirming the interest of such approach.

## Introduction

Low grade inflammatory tone associated to obesity displays a causal role in insulin resistance and metabolic syndrome [1,2]. Adipose tissue expansion during obesity plays a key role in the genesis of such inflammatory status. Indeed, adipocytes are able to express chemokines involved in the attraction of most immune cells [3], but also a large range of cytokines [4]. In parallel to the development of low-grade inflammation, overweight and obesity are also associated to reduced plasma levels and/or adipose concentrations of micronutrients, including retinol [5,6] and metabolites of vitamin D (VD) [7,8]. Hence, these insufficiencies are suspected to influence the development of inflammation and insulin resistance [5,9–11]. Indeed, several studies have reported an anti-inflammatory effect of all-trans retinoic acid (ATRA) [3,12–16] and 1,25(OH)_2_D on adipocytes [17–22].

In addition to the contribution of cytokines and chemokines in the inflammatory status, microRNAs (miRNAs) are now considered as key actor in inflammatory mediation [23]. MicroRNAs are endogenous, noncoding, and single-stranded RNAs of 22 nucleotides and constitute a class of gene regulators [24]. The mature miRNAs regulate gene expression depending on the degree of complementarity between the miRNA and its target. Indeed, miRNAs that bind to the 3’ UTR of mRNA with imperfect complementarity block protein translation, while miRNAs that bind to mRNA with perfect complementarity induce targeted mRNA cleavage [25]. The miRNAs are involved in the control of many cellular processes, such as cell differentiation, growth, proliferation, and apoptosis [26]. Many studies have reported that they have also been associated with physiopathological disorders related to obesity, such as oxidative stress, impaired adipogenesis, insulin signalling, angiogenesis, and inflammation [23,27–29]. Furthermore, some of them are secreted in vesicles and actively participate in intercellular communication [30–32]

The objective of the present work was first to unveil the effect of ATRA on miRNA expression in adipocytes and adipose tissue. In particular, we focused our attention on miR-146a, 150, and 155 that were involved in inflammation, obesity-related diseases, and adipocyte function [33,34]. Then, based on previous results on inflammatory effects of ATRA and 1,25(OH)_2_D, we wanted to assess bioinformatically, through mRNA and miRNA levels, common pathways impacted by ATRA and 1,25(OH)_2_D that modulate the overall transcriptomic response in adipocytes.

## Material and methods

**Cell culture** - Adipocyte cells were grown at 37°C in a 5% CO_2_ humidified atmosphere. The human preadipocytes (three independent cultures) were obtained from Promocell (Heidelberg, Germany) and cultured according to the manufacturer’s instructions. At confluence, preadipocytes were differentiated with differentiation medium (Promocell) for 72 h. Then, cells were maintained in nutrition medium (Promocell) until the obtention of mature adipocytes (day 12). Next, adipocytes were incubated with ATRA (2 µM, 24 h) followed by a 24-h incubation with TNFα (15 ng/mL). Experiments were performed in triplicate, on three independent cultures.

The 3T3-L1 preadipocytes (ATCC, Manassas, VA) were seeded in 3.5 cm-diameter dishes at a density of 15 × 10 [4] cells/well, and grown in DMEM supplemented with 10% FBS, at 37°C, as previously reported [35]. To induce differentiation, 2-d post-confluent 3T3-L1 preadipocytes (day 0) were stimulated for 72 h with 0.5 mM isobutylmethylxanthine, 0.25 µmol/L dexamethasone, and 1 µg/mL insulin in DMEM supplemented with 10% FBS. The cultures were then treated with DMEM supplemented with 10% FBS and 1 µg/mL insulin. The mature adipocytes (day 8) were incubated with ATRA (2 µM, 24 h) followed by a 24-h incubation with Escherichia coli lipopolysaccharide (LPS) (1 ng/mL; serotype O111:B4, Sigma-Aldrich). Experiments were performed in triplicate.

**Animal experiment** - The protocol was approved by the local ethics committee. Six-week-old male C57BL/6 J mice were obtained from Janvier (Le Genest-Saint-Isle, France). Mice were fed ad libitum (chow diet A04, Safe, Augy, France), with full access to drinking water. The animals were maintained at 22°C under a 12 h light–12 h dark cycle at 50% humidity. To assess the impact of ATRA on acute inflammation, the mice received by gavage (*n* = 6–9 per group) ATRA (5 mg/kg of body weight; Sigma-Aldrich, Saint-Quentin-Fallavier, France) or vehicle alone (olive oil), once a day for 4 d. On the fifth day, the mice were injected intraperitoneally with saline or Escherichia coli LPS (4 mg/kg; serotype O111:B4, Sigma-Aldrich). The mice were sacrificed 4 h after LPS injection, and epididymal adipose tissue was dissected and stored at −80°C.

**Extracellular vesicles (EVs) isolation** - EVs were extracted by using miRCURY Exosome Cell/Urine/CSF kit (Qiagen, Courtaboeuf, France) according to manufacturer’s instructions for total EVs.

**RNA isolation and qPCR** - Total cellular RNA was extracted using TRIzol reagent, according to the manufacturer’s instructions. miRNAs from the supernatant and EVs were extracted with miRNeasy Serum/Plasma kit (Qiagen, Courtaboeuf, France) according to manufacturer’s protocol. For total extracellular miRNA, 200 µL of conditioned media was used as the starting material. For the total EVs, 800 µL of resuspended EVs was used to extract miRNAs.

To quantify miR-146a, miR-150, and miR-155 in cells and tissues, cDNAs were first synthesized from 1 µg of total RNA in 20 μL using 5× miScript Hispec Buffer, 10× nucleic mix, and miScript reverse transcriptase according to the manufacturer’s instructions (Qiagen, Courtaboeuf, France). For extracellular miRNAs, cDNAs were synthesized from 10 µL of miRNAs in the same reaction mix as total cellular RNA.

Real-time quantitative RT-PCR analyses were performed using the M×3005P Real-Time PCR System (Stratagene, La Jolla, CA), as previously described [36]. Reactions were performed in a 12.5 μL volume containing 6.25 μL of 2× QuantiTect SYBR Green PCR Master Mix (Qiagen, Courtaboeuf, France), 1.25 μL of 10× miScript Universal Primer (Qiagen, Courtaboeuf, France), 1.25 μL of 10× miScript Primer Assay [Mm_miR146_1 miScript Primer Assay, Mm_miR-150_1 miScript Primer Assay, Mm_miR-155_1 miScript Primer Assay, Hs_SNORD68 miScript Primer Assay (Qiagen, Courtaboeuf, France), Hs_RNU6-2_1 miScript Primer Assay (Qiagen, Courtaboeuf, France) and Ce_miR-39_1 (Qiagen, Courtaboeuf, France)] and 2.5 μL of RNase-free water. For each condition, the expression was quantified in duplicate. The SNORD68 and RNU6 were used as the endogenous control for cells in the comparative cycle threshold (C_T_) method. For miRNAs from EVs, Cel-39 was used as an external spike used for qPCR normalization.

**miRNA PCR arrays** - The miRNA PCR arrays (Qiagen, Courtabœuf, France) were used to quantify miRNAs extracted from human culture adipocytes, according to the manufacturer’s instructions. Reactions were performed in a 12.5 μL volume containing 6.25 μL of 2× QuantiTect SYBR Green PCR Master Mix (Qiagen, Courtabœuf, France), 1.25 μL of 10× miScript Universal Primer (Qiagen, Courtabœuf, France). After an initial incubation step of 15 min at 95°C, amplification reaction was performed in 40 cycles comprising 3 steps (94°C, 15 s; 55°C, 30 s; and 70°C, 30 s). For each condition, the expression was quantified in duplicate and the SNORD68 or RNU6 were used as endogenous control in the comparative cycle threshold (C_T_) method.

**Bioinformatic analysis** - The data from microarrays were analysed with MetaCore and Ingenuity Pathway Analysis softwares which allow to identify metabolic pathways predicted from an expression gene list. We also used TargetScan software to predict biological targets of miRNAs. All data are available upon request from the corresponding author.

**Statistical analysis** - The data are expressed as the means ± SEM. Significant differences between the control and treated groups were determined using ANOVA, followed by the PLSD Fischer post hoc test using Statview software, and *p* < 0.05 was considered statistically significant.

## Results

### All-*trans*-retinoic acid modulates miRNA expression in mouse adipose tissue and adipocytes as well as in human adipocytes

**Figure 1.**
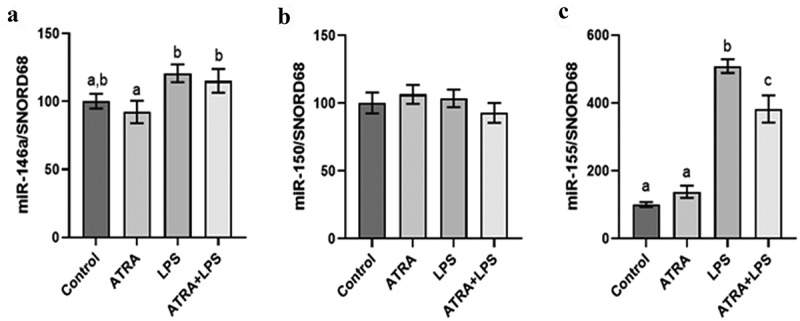
ATRA modulates miRNA expression levels in mouse adipose tissue. Effect of ATRA (5 mg/kg) and LPS (4 mg/kg) on the levels of miR-146a (a), miR-150 (b) and miR-155 (c) in mice adipose tissue compared with control (olive oil). The miRNA levels were quantified by miRNA qPCR. SNORD68 were used as the endogenous control. The values are presented as the means ± SEM. Bars not sharing the same letters are significantly different, *p* < 0.05.

**Figure 2.**
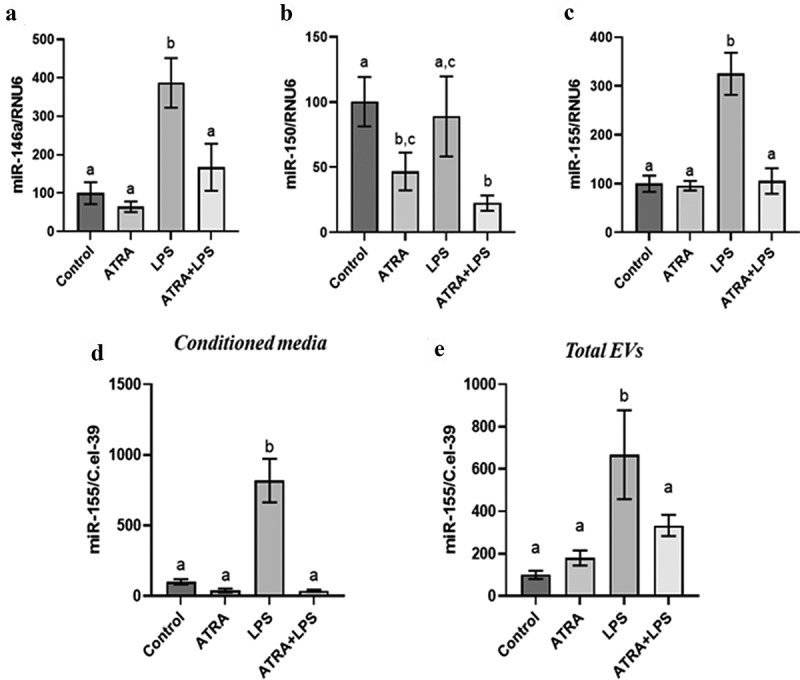
ATRA modulates miRNA expression levels in 3T3-L1 murine adipocytes and in EVs. Effect of ATRA (2 µM) and LPS (1 ng/mL) on the levels of miR-146a (a), miR-150 (b) and miR-155 (c) in 3T3-L1 murine adipocytes and miR-155 in the supernatant (d) and extracellular vesicles (e) compared with control (DMSO). The miRNA levels were quantified by miRNA qPCR. RNU6 were used as the endogenous control for cells and C. el-39 was used as exogenous control for extracellular miRNAs. The values are presented as the means ± SEM. Bars not sharing the same letters are significantly different, *p* < 0.05.

Based on our previous results on 1,25(OH)_2_D [34], the modulation of miR-146a, miR-150, and miR-155 by ATRA was assessed in a mouse model of acute inflammation. In the adipose tissue, LPS injections induced intracellular miR-155, miR-146a but not miR-150 expression (Figure 1). In addition, ATRA treatment during 4 d in mice reduced LPS mediated induction of miR-155 in adipose tissue (Figure 1c). These results were confirmed in 3T3-L1 adipocytes where LPS induced the expression of miR-155 and miR-146a but failed to modulate miR-150 expression (Figure 2). ATRA preincubation significantly reduced the LPS-mediated induction of the miR-155 and miR-146a (Figure 2a–c). The extracellular miRNA expression was quantified in conditioned media and isolated total extracellular vesicles. Under LPS induction, only the expression of miR-155 was induced and ATRA preincubation significantly reduced its expression compared with LPS alone (Figure 2d,e).

**Figure 3.**
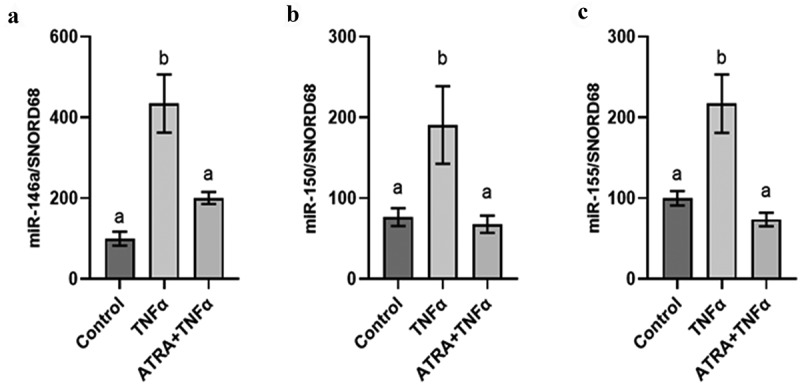
ATRA limits microRNA expression levels in human adipocytes. Effect of ATRA (2 µM) and TNFα (15 ng/mL) on the levels of miR-146a (a), miR-150 (b) and miR-155 (c) in human adipocytes compared with vehicle (DMSO). The miRNA levels were quantified by miRNA PCR arrays approach. SNORD68 were used as the endogenous control. The values are presented as the means ± SEM. Bars not sharing the same letters are significantly different, *p* < 0.05.

The impact of ATRA on the modulation of miRNAs was also assessed in human adipocytes submitted to inflammatory stress. To this aim, primary cultures of human adipocytes were preincubated with ATRA for 24 h followed by incubation with TNFα. This approach allowed us to quantify 84 miRNAs and 6 housekeeping genes (data not shown). The analysis of results has shown that 76 miRNAs were expressed in adipocytes (data not shown). Among these miRNAs, only miR-146a, miR-150, and miR-155 were positively regulated by TNFα (Figure 3). Interestingly, their expression decreased in adipocytes preincubated with ATRA in inflammatory conditions compared with TNFα condition (Figure 3).

## Canonical pathways affected by mRNA and miRNA target genes under ATRA and 1,25(OH)_2_D converge to NF-κB signalling

In previous studies, we showed that ATRA [16] and 1,25(OH)_2_D [21] limited chemokine expression in human adipocytes. Data have shown that 3601 genes were regulated by ATRA in inflammatory conditions. Among these genes, 956 were positively regulated and 2645 were negatively regulated (Fold Change (FC: 1.5)). 5505 genes were regulated by 1,25(OH)_2_D. Among these genes, 833 were positively regulated and 4672 were negatively regulated (FC: 1.5) (Table 1).

**Table 1.** Genes regulated by ATRA and 1,25(OH)_2_D in inflammatory conditions.

	ATRA+TNFα	1,25(OH)_2_D+TNFα
Number of genes regulated (FC 1.5)	3601	5505
Number of genes positively regulated (FC 1.5)	956	833
Number of genes negatively regulated (FC 1.5)	2645	4672

FC : Fold change.

ATRA (present study) and 1,25(OH)_2_D [34] modulated the expression of the same miRNAs in human adipocytes (miR-155, miR-146a, and miR-150).

Using TargetScan software, putative target genes of the three miRNAs were predicted (Supplemental Tables 1, 2 and 3). It appeared major differences in the number of genes targeted by each miRNA. Indeed, 200 genes are potentially targeted by miR-146a, 275 by miR-150, and 440 by miR-155 (Table 2).

**Table 2.** Genes targeted by miR-146a, miR-150, and miR-155.

	miR-146a	miR-150	miR-155	All miRNAs
Number of genes targeted by miRNAs (Target Scan)	200	275	440	874
Number of canonical pathways impacted by miRNAs (MetaCore)	0	28	140	201

**Table 3.** A combination of miRNA PCR array and mRNA microarray data.

	ATRA+TNFα	1,25(OH)_2_D+TNFα
Number of putative target genes (/Target Scan)	46	45
% of predicted genes (/Target Scan)	5.20%	5.1%

**Figure 4.**
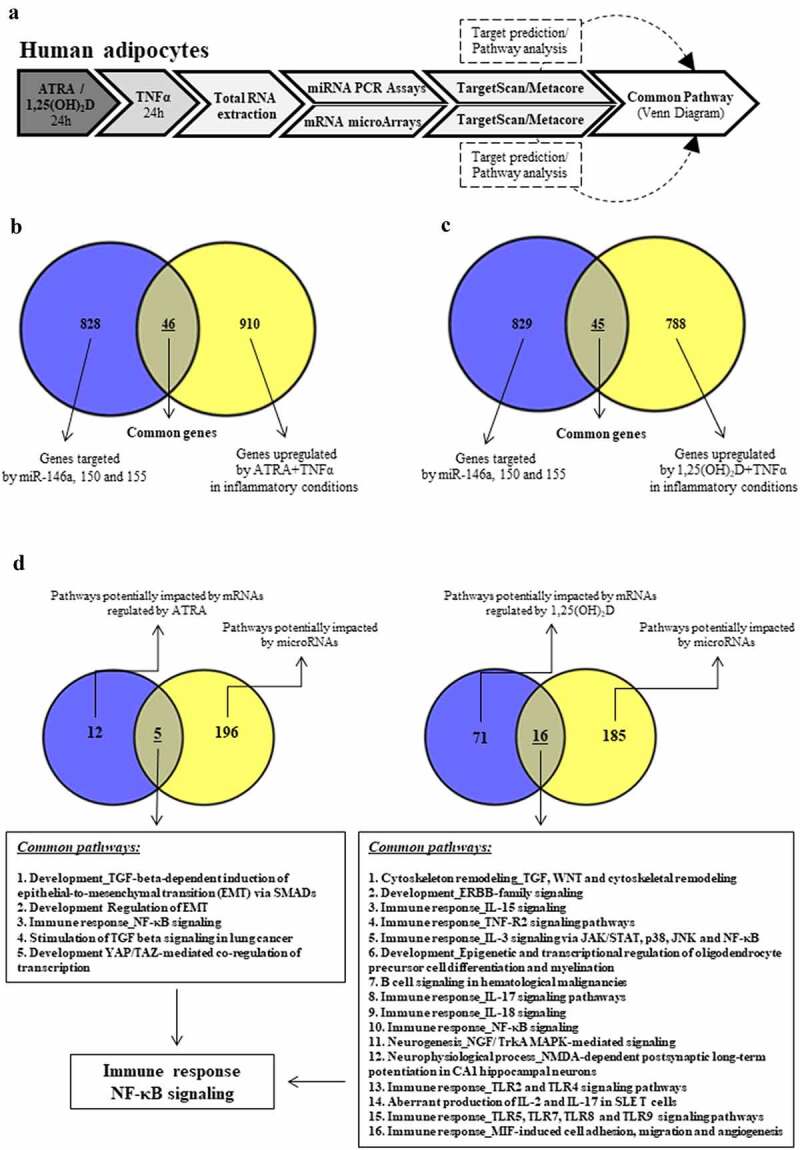
Metabolic pathways affected by mRNA and miRNA target genes converge to NF-κB signalling. a. Workflow from the human adipocytes culture to data analysis. b. Common genes regulated by ATRA or 1,25(OH)_2_D in inflammatory conditions and genes potentially targeted by miRnas (Venn diagram). c. Common pathways regulated by ATRA or 1,25(OH)_2_D in inflammatory conditions and genes potentially targeted by miRNAs (Venn diagram). d. The signalling NF-κB pathway appeared to be impacted by both ATRA and 1,25(OH)_2_D.

To evaluate the global impact of ATRA and 1,25(OH)_2_D on adipocyte transcriptome and overlaps in terms of impacted biological and signalling pathways, we combined results of mRNA microarrays analysed in our previous studies [16,21] and results generated using miRNA PCR arrays in the present study and in previous study reporting the effect of 1,25(OH)_2_D [34]. Indeed, with the Venn diagram, we identified a set of 46 genes that were both upregulated by ATRA in inflammatory conditions (Figure 4b) and putatively targeted by miR-146a, miR-150, and miR-155. A similar analysis was conducted for 1,25(OH)_2_D and 45 genes appeared to be upregulated in both the mRNA list and putative miRNA target genes (Figure 4c) (Table 3).

After this analysis was performed at the gene level, we conducted a similar analysis at canonical pathway levels using MetaCore software. Data analysis showed that 28 pathways were regulated by miR-150 and 146a pathways by miR-155 (Table 2). In parallel, we identified canonical pathways potentially impacted by ATRA and 1,25(OH)_2_D. Thus, 17 pathways were regulated by ATRA and 87 by 1,25(OH)_2_D.

Finally, we combined the results of the bioinformatic analysis conducted in miRNA PCR array and mRNA microarray approaches. Using a Venn diagram, we combined the list of canonical pathways affected by miR-146a, miR-150, and miR-155 and those by mRNAs in inflammatory conditions. Five canonical pathways were found to be commonly affected by the three miRNAs and by mRNAs regulated by ATRA. Sixteen pathways were commonly affected in the case of 1,25(OH)_2_D (Figure 4d). Among these common pathways, only one pathway (Immune response_NF-κB signalling) appeared to be common between the two lists (Figure 4d).

## Discussion

In the present study, we demonstrated that ATRA was able to modulate the expression of inflammatory-induced miRNAs in adipocytes and in adipose tissue. If our group has already evidenced the impact of 1,25(OH)_2_D on miRNA expression in inflamed adipocytes [34], no studies have been reported on the impact of VA or active metabolite on miRNA expression in this context. MicroRNA-146a, miR-150, and miR-155 that we identified in our study as upregulated by TNFα or LPS have been implicated in the regulation of inflammation in adipocytes, but also in many other tissues or cell types [37,38]. Indeed, miR-146a is a typical miRNA involved in the control of the inflammatory response of innate immune system cells and in particularly monocytes/macrophages [39]. It was found to be induced in human monocytes under LPS stimulation in a NF-κB-dependent manner [40]. In several inflammatory contexts, including diabetic nephropathy, miR-146a displayed an anti-inflammatory effect, since miR-146a deficiency during diabetes led to increased expression of M1 activation markers and proinflammatory cytokines (IL1β, IL18) and suppression of M2 markers in macrophages [41]. In the obesity context, it has recently been demonstrated that miR-146a^−/−^ mice gained significantly more body weight when subjected to a high fat diet and increased fat mass, insulin-resistance, liver steatosis, and glucose intolerance, suggesting that miR-146a is able to regulate systemic and adipocyte glucose homoeostasis [42] and promote suppression of pro-inflammatory signalling pathways. The fact that our results showed that ATRA reduced the inflammatory-mediated induction of miR-146a could thus participate to the equilibrium of pro- and anti-inflammatory responses mandatory to achieve inflammatory control.

MiRNA-150 has been reported to be mandatory for B cell development and suppressed obesity-associated inflammation via regulation of B-cell function in adipose tissue [43,44]. Interestingly, miR-150^−/−^ mice displayed lower body weight and improved glucose homoeostasis as well as many other metabolic benefits accompanied to reduced body weight, suggesting that this mir-150 could represent an interesting target or biomarker regarding obesity and insulin resistance [45]. Thus, the observed reduction of miR-150 under ATRA treatment could participate to beneficial anti-inflammatory effect of the VA metabolite, at least in human adipocytes.

In the present study, we also reported an induction of miR-155 under inflammatory stimulation. Such induction is fully consistent with the literature where it has been described that miR-155 expression has been associated with inflammatory response and diseases [46]. Furthermore, in a previous study, we have demonstrated that miR-155 is induced in murine and human adipocytes in inflammatory conditions through TNFα, via an NF-kB-dependent mechanism [33]. Thus, the inhibitory effect of ATRA regarding the expression of mir-155 could be a key element of the anti-inflammatory effect of ATRA. We also previously reported that inflammation could enhance miR-155 expression in the supernatant of adipocytes in culture [33], suggesting that inflamed adipocytes could secrete EVs containing miR-155. To support such hypothesis, in the present study, we confirmed that inflammation increased extracellular miR-155 in the supernatant but also in purified EVs. Interestingly, ATRA was able to reduced such miR-155 induction in the supernatant and in EVs. Since EVs containing miRNAs are known to vehiculate information between cells and regulate gene expression in other tissues [47], we could hypothesize that ATRA, via a down-regulation of miRNA content, may reduce adipose tissue-related inflammatory tone in various cell types. The influence of those miRNAs, contained in adipocyte-derived-EV, on target cells remains to be elucidated, but this may help to determine the whole-body effect of the vitamins. Such assumptions will require further experimentation.

Altogether, the reported effects of ATRA on the expression of several miRNAs related to inflammation are strongly in agreement with previous reports, showing that ATRA displayed anti-inflammatory effect via reduced expression of cytokines or chemokines by adipocytes or adipose tissue [3,12–16]. It is noteworthy that similar results have already been reported with 1,25(OH)_2_D at both the cytokine level [17–22] and the miRNA level [34]. Thus, we wanted to take advantage of these data to evaluate the possibility of a common mechanism of action, shared by ATRA and 1,25(OH)_2_D, to mediate anti-inflammatory effects in adipocytes. To this aim, we implemented a bioinformatic combination of transcriptomics data (both mRNA and miRNA) generated in primary cultures of human adipocytes.

The combination of the canonical pathways affected by miR-146a, miR-150, and miR-155 and those by mRNAs in adipocytes incubated with ATRA or 1,25(OH)_2_D followed by inflammatory stress have shown that canonical pathways affected by mRNA and miRNA target genes converge to NF-κB signalling. Such identification of the NF-κB pathway as a common driver of ATRA and 1,25(OH)_2_D is particularly relevant because it had been shown that this signalling was able to mediate a large part of the inflammatory response in adipocytes [48–50]. We have also shown that this pathway was involved in miR-155 regulation by TNFα in adipocytes submitted to inflammatory stress [33] as well as in ATRA anti-inflammatory effect [16] and 1,25(OH)_2_D effect [18]. Indeed, these two molecules displayed a strong inhibitory effect on NF-κB signalling in 3T3-L1 adipocytes characterized by a reduction of the phosphorylation levels of IκB and p65, two main proteins of the NF-κB signalling pathway [16,21].

Thus, our results demonstrate that ATRA and 1,25(OH)_2_D seem to have similar effects on inflammatory markers and similar molecular mechanisms involved in obesity-associated inflammatory response. Furthermore, it confirms the interest of such approach based on a combined analysis of gene sets and miRNA sets to highlight similarities in terms of molecular mechanisms involved in the overall transcriptomic response. Indeed, in the present case, the result generated *i.e*. the involvement of NF-kB signalling has been largely experimentally validated as previously mentioned. However, such approach has some limitations and can be considered only as informative. Indeed, it is largely based on miRNA target gene prediction tools, which are far from perfect, and results may be highly variable depending on the choice of parameters used to conduct the analysis.

In conclusion, the present study reported that ATRA regulated inflammation-linked miRNA not only in adipocytes and adipose tissue but also in adipocyte-derived EVs. Such data reinforce our knowledge related to the health effects of lipophilic vitamins. Furthermore, the integrative network-based analysis of mRNA and miRNA expression in adipocytes represents a new approach for data analysis that should help to identify common molecular mechanisms.

## Supplementary Material

Supplemental Material

## Data Availability

The data that support the findings of this study are available from the corresponding author, JFL, upon reasonable request.
